# Brusatol, an NRF2 inhibitor for future cancer therapeutic

**DOI:** 10.1186/s13578-019-0309-8

**Published:** 2019-06-06

**Authors:** Sabrina J. Cai, Yang Liu, Sue Han, Chunzhang Yang

**Affiliations:** Neuro-Oncology Branch, Center for Cancer Research, National Cancer Institute, NIH, Building 37, Room 1142E, Bethesda, MD 20892 USA

**Keywords:** Brusatol, Cancer, NRF2, Oxidative stress, Synthetic lethality

## Abstract

**Background:**

Natural products from herbal medicines have long been investigated for their potentials as cancer therapeutics. Besides the development of several herbal medicine-derived anti-cancer agents, such as paclitaxel, vincristine and podophyllotoxin, many recent laboratory findings demonstrated that brusatol, a quassinoid from the seeds of *Brucea sumatrana*, exhibits potent tumor suppressing effect with improved disease outcome. Our recent finding further demonstrated that brusatol synergizes with the intrinsic metabolic burden in cancer cells.

**Main body:**

Here, we summarized the recent investigations of brusatol as an experimental therapeutic for human malignancies, such as leukemia, lung cancer, pancreatic cancer and brain tumor. We also discussed the molecular target brusatol, with a focus on the Nuclear factor erythroid 2-related factor 2 (NRF2)-guided gene transcription, as well as glutathione de novo synthesis. Further, we discussed the challenges and future applications of brusatol for cancer therapy.

**Conclusion:**

In conclusion, we believe increasing evidences have shown the value of brusatol as a novel strategy for cancer treatment, which may indicate future drug development and clinical translation.

## Introduction

The quassinoid brusatol was first isolated and characterized from the seeds of *Brucea sumatrana* in 1968. Despite its known application as a treatment for amebiasis, brusatol has been rapidly recognized as a potent anticancer agent through the past few decades. However, there has long been arguments regarding the mechanism and specificity of brusatol, which limits the further developments and proceeding of this compound into clinical practice. In this article, we discussed recent discoveries of brusatol as an experimental therapeutic for human malignancies. We also discussed the latest findings of the molecular targets of brusatol, as well as its value as a future cancer therapeutic.

### Application of brusatol as an experimental cancer therapeutic

Brusatol was firstly evaluated as an experimental therapeutic for leukemia. In 1979, Hall et al. [1] used a P-388 lymphocytic leukemia model to evaluate the biologic impact of five quassinoids. Both in vitro and in vivo findings showed that brusatol exhibits potent suppression on tumor cell metabolism and proliferation. The cell cycle arrest, cytotoxicity and terminal differentiation in leukemia cells were later verified by an investigation using multiple leukemia cell lines, with remarkably down-regulation of c-myc [2]. Besides the investigations on hematopoietic malignancies, several pioneering findings indicated that brusatol is effective to epithelial types of tumors. For example, Ren et al. [3] discovered that brusatol established synergistic cytotoxicity with first line lung cancer treatment cisplatin, which reduced A549 cancer cell oncogenesis. A follow-up study further indicated that brusatol is effective to suppress gefitinib-resistant lung cancer cells and xenografts [4]. Similarly, an in vitro investigation on pancreatic cell line PATU-8988 and PANC-1 showed that brusatol monotherapy resulted in substantial cytotoxicity in these cells [5]. A follow-up study by the same team showed that the combining brusatol enhanced the therapeutic effect of gemcitabine, evidenced by enhanced apoptotic changes and diminished xenograft formation [6]. An investigation of colorectal cancer showed that brusatol reduced xenograft expansion, with suppressed expression of HIF-1α and c-myc. Accordingly, our recently investigation showed that brusatol resulted in potent tumor suppression in *IDH1*-mutated glioma, through disrupting the redox homeostasis and overwhelmed oxidative damage [8].

### Brusatol as NRF2 inhibitor

**Fig. 1:**
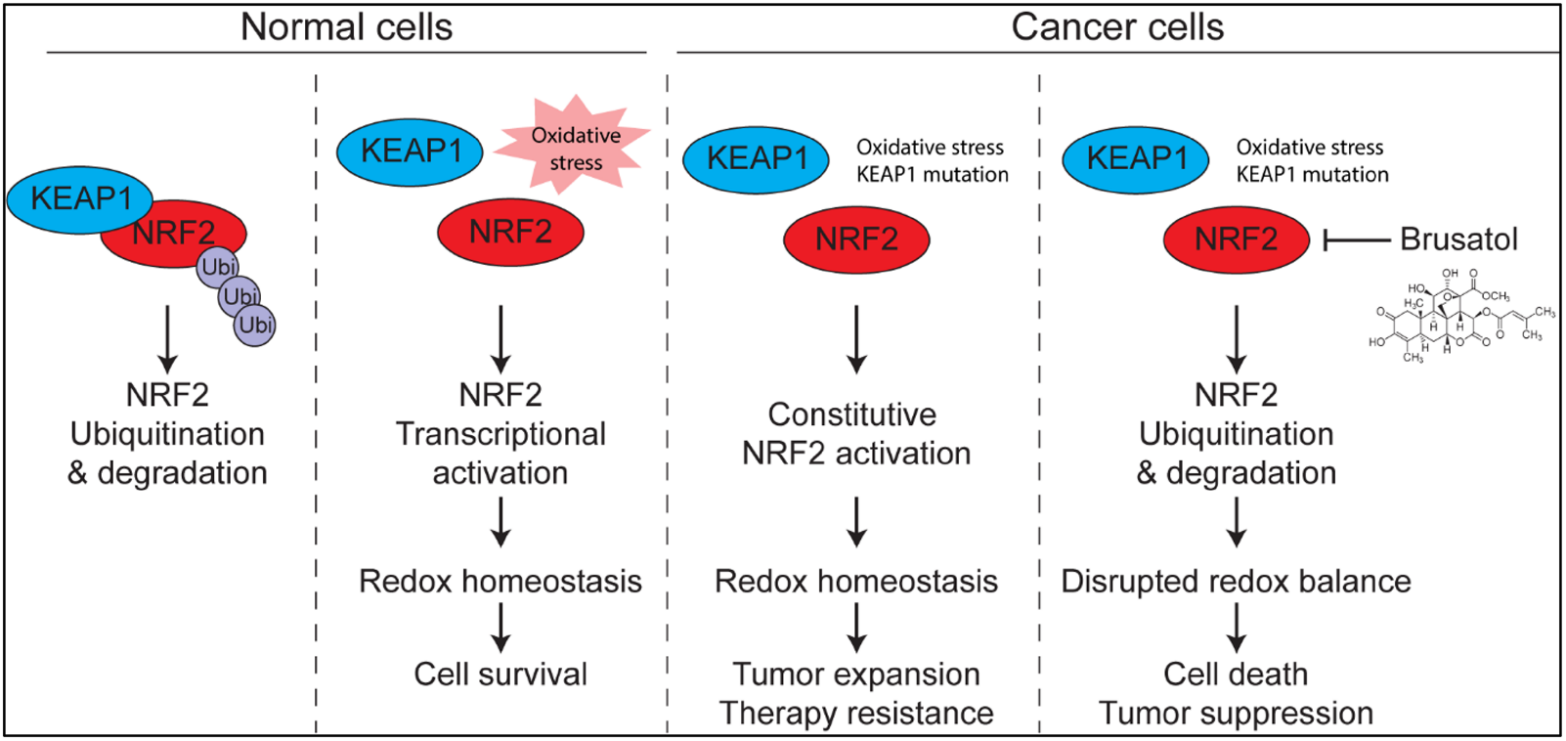
Schematic illustration of brusatol anticancer effect by inhibiting NRF2 activity. NRF2 is the key transcription factor regulating oxidative homeostasis. In normal cells, NRF2 is recognized by KEAP1 for ubiquitination and degradation. Oxidative stress compromises the function of KEAP1, allowing the stabilization and transcriptional activity of NRF2. The expression of NRF2 downstream genes maintain the oxidative homeostasis and cell survival. Cancer cells tend to exhibit constitutive NRF2 activity through intrinsic oxidative stress and KEAP1 mutations, and therefore support tumor expansion and therapy resistance. Brusatol inhibits NRF2 by enhancing protein ubiquitination, resulting in the disrupted redox balance, cell death and tumor suppression

Despite the robust anti-tumor activity, the molecular target of brusatol has long been elusive. In 2011, Ren et al. [3] firstly described that NRF2-mediated defense mechanism is suppressed by brusatol. NRF2 is a multi-function transcription factor that governs multiple pathways, such as glutathione synthesis, ROS scavenging, drug excretion and detoxification, and NADP synthesis. Under physiological conditions, NRF2 protein is recognized by its E3 ligase KEAP1, and therefore proceeds to ubiquitination and proteasomal degradation. Metabolic stress, such as exposure to ROS, compromises the function of KEAP1, which results in the stabilization of NRF2 protein. Thus NRF2 translocates into the cell nucleus and initiate transcriptional activation to support cellular metabolism and survival. For example, NRF2 enhances the transcription of glutamate-cysteine ligase (encoded by *GCLC* and *GCLM*) and cystine/glutamate transporter (encoded by *SLC7A11*), which facilitate the internalization of cystine to support glutathione de novo synthesis. Glutathione serves as an antioxidant, preventing oxidative damage to the important cellular components such as DNA, lipid and proteins. Cancer cells, on the other hand, tend to exhibit constitutive NRF2 activation, due to genetic abnormalities in KEAP1 or intrinsic metabolic deficiencies. NRF2 downstream genes serve as key protective mechanism for cancer cells, granting metabolic privilege and survival advantage throughout oncogenesis (Fig. 1).

### Current challenges and future directions

With the expansion of basic/translational investigations, the tumor suppressive effect of brusatol has been highlighted in a variety of cancer types. However, there are several issues that limit the proceeding of this compound into clinical application. Firstly, the specificity of brusatol is still unclear. While most studies reported brusatol as an NRF2 inhibitor [3], there are several reports indicated different mechanisms, including direct inhibition of protein synthesis [9], or down-regulation of c-myc [2, 7]. A bioinformatic study predicted that 464 proteins could be potentially targeted by brusatol. Although this hypothesis has not been validated through biological studies, it implied that off-target could be an issue for the future applications of brusatol [10]. Crystallography study resolving brusatol-NRF2 interaction, as well as optimization of brusatol molecular structure could be promising approaches to improve the specificity for NRF2 targeting. Secondly, brusatol exhibits systemic toxicity. Severe side effects have been observed through early phase clinical studies, which includes hypotension, nausea and vomiting. Thus, the dosage of brusatol should be carefully justified. Moreover, optimizing brusatol specificity to NRF2 may be helpful to reduce side effect, which is currently in urgent need for future clinical applications.

## Conclusion

Collectively, these evidences revealed that brusatol is a promising anticancer compound by disrupting redox homeostasis. Brusatol serves as a sensitizer when combined with other anticancer regimens. Monotherapy of brusatol could be effective for certain types of cancers that have high burden of oxidative stress. Although targeting specificity and systemic toxicity remain an issue, numerous findings have shown that targeting NRF2 defensive mechanism could be a novel therapeutic strategy for human malignancies.

## Abbreviations

NRF2: nuclear factor erythroid 2-related factor 2
HIF-1α: hypoxia-induced factor-1α
IDH1: isocitrate dehydrogenase 1
ROS: reactive oxygen species
NADP: nicotinamide adenine dinucleotide phosphate
KEAP1: kelch like ECH associated protein 1
